# Research Progress on Circular RNA in Glioma

**DOI:** 10.3389/fonc.2021.705059

**Published:** 2021-10-21

**Authors:** Mengyu Chen, Chunyan Yan, Xihe Zhao

**Affiliations:** Department of Clinical Oncology, Shengjing Hospital of China Medical University, Shenyang, China

**Keywords:** circRNA, noncoding RNA, glioma, oncogenesis, metabolism

## Abstract

The discovery of circular RNA (circRNA) greatly complements the traditional gene expression theory. CircRNA is a class of non-coding RNA with a stable cyclic structure. They are highly expressed, spatiotemporal-specific and conservative across species. Importantly, circRNA participates in the occurrence of many kinds of tumors and regulates the tumor development. Glioma is featured by limited therapy and grim prognosis. Cancer-associated circRNA compromises original function or creates new effects in glioma, thus contributing to oncogenesis. Therefore, this article reviews the biogenesis, metabolism, functions and properties of circRNA as a novel potential biomarker for gliomas. We elaborate the expression characteristics, interaction between circRNA and other molecules, aiming to identify new targets for early diagnosis and treatment of gliomas.

## 1 Introduction

Glioma is one of the most common tumors in central nervous system (CNS). It is highly malignant and difficult to remove safely. Even with comprehensive treatment (i.e., surgery, radiotherapy and chemotherapy), 90% of patients relapse (1). The pathogenesis of glioma remains unclear. Finding the cause of glioma could provide new strategies for screening, diagnosing and treating this disease. Abnormal gene expression is present in gliomas, including altered expression of high amounts of coding and non-protein encoding RNAs, activation of oncogenes and/or deactivation of cancer suppressor genes.

Of the entire human genome, only a small part encodes proteins. About 98% of the genome is transcribed as non-coding RNA (ncRNA), such as long non-coding RNA (lncRNA), circular RNA (circRNA), microRNA (miRNA) and piwiRNA. Non-coding RNAs were initially identified as alternative splicing errors that could not be translated into protein, but as the research progressed, circRNA was found to encode peptides. With the advent of bioassay technology, increasing ncRNAs were discovered to regulate and perform multiple biological functions at the RNA level (2). CircRNA is a novel single-stranded ncRNA with an estimate of exceeding 100,000 different circRNAs in the human transcriptome (3–5), and the majority of them are endogenous RNAs. In human cells, circRNA is common, sometimes ten times more abundant than their corresponding linear mRNAs (6). CircRNA expression is spatiotemporal-specific and conserved among species, which is promising to become ideal biomarkers for cancer diagnosis (7). Moreover, circRNA may modulate the expression of cancer-related genes or participate in regulating oncogenic mechanisms and regulatory pathways. This article mainly reviews the biogenesis, functions of circRNA and its emerging roles in glioma pathogenesis and clinical treatment.

## 2 An Overview of circRNA

### 2.1 Biogenesis and Localization

**Figure 1:**
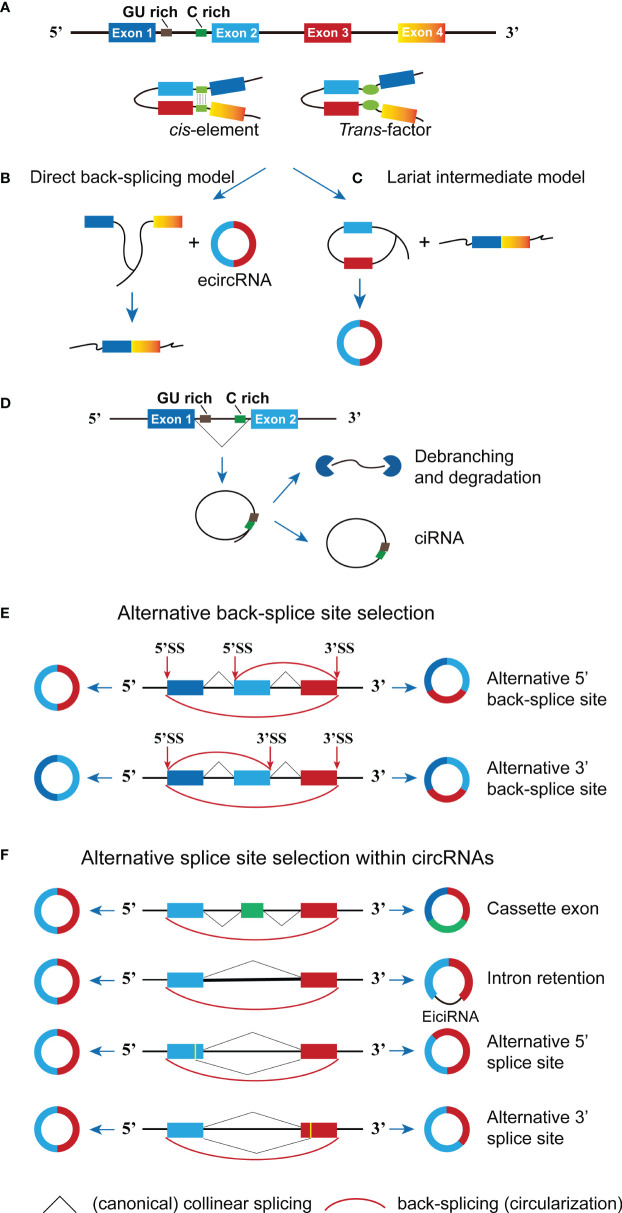
The formation of circRNA. **(A)** The *cis* sequence and *trans* factor located in the intron region causes the intron region to pair and promote circRNA generation. **(B)** In the direct back-splicing model, back-splicing occurs first and circRNA along with an exon-intron lariat intermediate is produced, the latter is further spliced to form linear RNA. **(C)** In the lariat intermediate model, splicing event occurs first and linear RNA is generated, followed by a long intron containing exons for later back-splicing to form circRNA. **(D)** The conserved sequences (GU rich and C rich elements) at both ends of the intron help the intron avoid being hydrolyzed by the lasso debranching enzyme to form ciRNA structures. **(E)** Two types of alternative back-splicing. **(F)** Schematic diagrams of four basic types of alternative splicing. The pictorial representation is not to scale.

The maturation of mRNA is an integrated process consisting of three main modifications: 5’ capping, 3’ polyadenylation and RNA splicing. CircRNA-forming exons are generated by an unusual alternative splicing mechanism termed back-splicing, in which the 3’-end of an exon is ligated to the 5’-end of its own or an upstream exon through a 3’,5’-phosphodiester bond. It has been reported back-splicing is catalyzed by the canonical spliceosomal machinery and could be regulated by both *cis* and *trans* regulatory factors (8) (**Figure 1A**). To be specifically, the reverse complementary sequence located in flanking introns may lead to intron pairing, thereby mediating reverse splicing to form a loop. This circularization could be mediated by base pairing between inverted repeat elements (e.g., *Alu* repeats) or through the binding of RNA binding proteins (RBP) to specific motifs located in the intron region. Interestingly, *cis* elements and *trans* factors could regulate circRNA biogenesis in a cooperative manner, several RBPs have been reported to regulate circRNA biogenesis through interaction with inverted repeated *Alu* pairs (IR*Alu*s) in human flanking introns (9, 10).

Currently, two models of circRNA biogenesis have been proposed based on the order of splicing events and different intermediates: direct back-splicing and lariat intermediate (11). As illustrated in **Figure 1B**, direct back-splicing model believes that back-splicing process happens first to produce a circRNA and a linear exon transcript, which could be subsequently spliced to form a linear mRNA. On the contrary, splicing event occurs earlier to generate a linear mRNA and a long intron lariat (containing exons) in the lariat intermediate model. The intermediate is further spliced to form circRNA composed of exons (**Figure 1C**). However, it remains unclear in which order steps occur and whether they could occur stochastically or synergistically, more studies are required to figure out specifics. CircRNAs can be grouped into three main categories given the richness and complexity of the genomic source regions, including exonic circRNA (ecircRNA), circular intronic RNA (ciRNA) and exon-intron circRNA (EIcircRNA) (12). The majority of endogenous circRNAs involve more than one exon, the average exon length in the multi-exonic circRNAs being shorter than those in the single-exonic circRNAs (average 112–130 *vs*. 353 nucleotides per exon) (13). The structure of the newly proposed *Saccharomyces cerevisiae* spliceosomal E complex provides a theoretical basis for this phenomenon. As shown in **Figure 1D**, the formation of ciRNAs depends on conserved sequences at both ends of the intron, which help the intron avoid being hydrolyzed by the debranching enzyme and form circular structures from intron lariats (14). Furthermore, the generation of EIcircRNA is possibly due to the lasso RNA formed by the introns fails to debranch normally and is not removed by further splicing (15) (**Figure 1F**).

Notably, diverse circRNAs can be generated from a single gene locus *via* back-splicing and alternative splice site selection (13). To be specifically, there are two types of alternative back-splicing, alternative 5’ back-splicing or 3’ back-splicing, in which the downstream 5’-end of an exon or upstream 3’ back-splice sites link to alternative 3’ or 5’ back-splice site through a 3’,5’-phosphodiester bond (**Figure 1E**). In addition, alternative splicing (i.e., cassette exons, intron retention, alternative 5′ splicing and alternative 3′ splicing) may also contribute to expanding the diversity of circRNAs (**Figure 1F**). CircRNA-specific cassette exons refer to exons that present in circRNAs but absent in the cognate mRNAs (13, 16). All in all, the accurate mechanism of alternative splicing within circRNAs is still unclear, and three types of circRNA molecules can be formed through the competitive complementary pairing between intron sequences (17). There is a competitive balance between these circRNA molecules, which can affect mRNA expression. Although the production efficiency of circRNA at the transcriptional level is low, it can gradually accumulate to higher expression levels through continuous production and minimal loss (18).

EIciRNA and ciRNA are generally localized to the nucleus, while ecircRNA is enriched in the cytoplasm (19). EcircRNA transport out of the nucleus may be mediated by DExH/D-box helicases and N6-methyladenosine modification (m6A). Huang et al. found that human UAP56 is necessary for the nuclear export of long circRNAs, while URH49 controls the localization of short circRNAs (20). Thus, the length of the mature circRNA may determine its nuclear exportation pathway. Furthermore, a recent study discovered that the m6A of circNSUN2 could modulate cytoplasmic transport by recruiting YTH domain-containing protein1 (YTHDC1) (21). Additionally, circRNAs can be transported through extracellular vesicles (EV) and is detectable in the circulation (22). Notably, a few studies published recently have shown that circRNAs can also be located in mitochondria (23, 24), which means that the derivation of circRNA is sophisticated and diverse and circRNA might also be located in other organelles or subcellular compartments.

### 2.2 Molecular Functions and Mechanisms

Research has found that even if the rate of gene transcription is artificially accelerated, it would still be difficult to increase circRNA production, indicating that circRNA is the product of fine-tuned cell regulation (25). Considerable studies have elucidated that some circRNAs participate in normal processes of cells or individuals through different molecular mechanisms, including serving as molecular sponges for microRNA (miRNA) and proteins, interfering with the normal splicing of RNA precursors and binding to RNA binding proteins [e.g., Argonaute (Ago), PolII and quaking (QKI)] that affect RNA transcription and can even be translated to polypeptides.

### 2.3 miRNA Sponges

**Figure 2:**
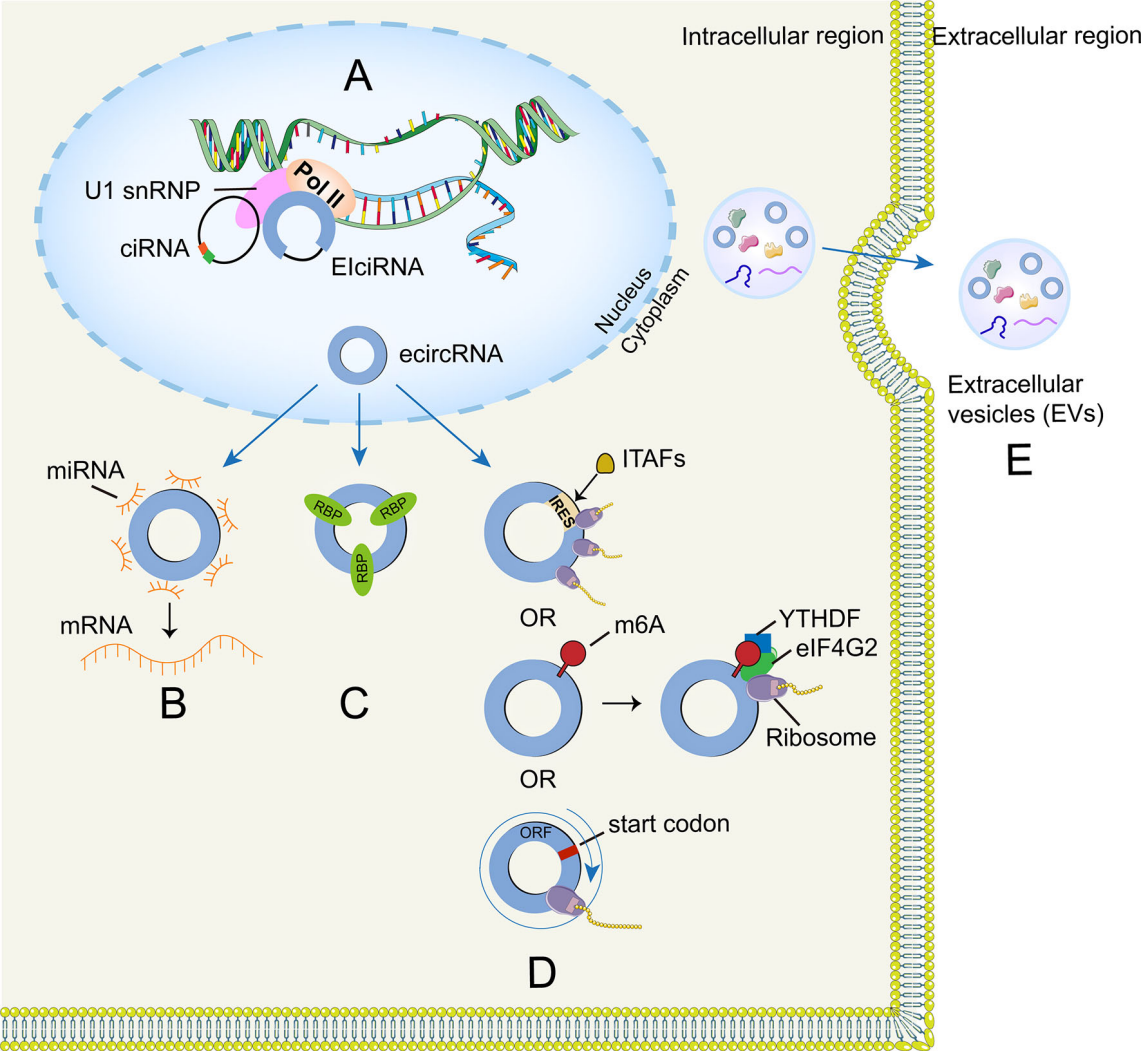
**|** Functions of circRNA. **(A)** CircRNA can combine with their host genes to modulate transcription and splicing. **(B)** CircRNA can function as miRNA sponges to regulate target gene expression. **(C)** CircRNA can form several interactions with proteins. **(D)** CircRNA can be translated through IRES-driven, m6A-driven mechanisms and rolling circle amplification mechanism. **(E)** Exosomal circRNA can be regarded as molecular biomarkers.

CircRNAs exert various biological functions in tumors, particularly as miRNA sponges (26) (**Figure 2B**). MiRNA is another type of ncRNA that is small, single-stranded with a length of about 21–25 nucleotides. Moreover, miRNA can associate with the 3’-untranslated region (3’-UTR) of a target mRNA to specifically inhibit target gene expression at the translational level in the form of RNA-inducing silencing complex (RISC) (27). CircRNAs often harbor more than one miRNA binding site and one miRNA can bind to several different circRNAs (17). For example, circFOXO3 could bind to miR-22, miR-136, miR-138 and miR-149 (28), while miR-1205 can bind to circPOSTN and circVPS18 (29, 30). CircRNAs that contain many miRNA response elements (MREs) could be competitively binding to miRNAs on the basis of complementary base pairing, resulting in the reduction in functional miRNAs levels that allows increased expression of target genes (**Figure 2B**). For instance, circSERPINE2 upregulates BCL2 expression (anti-apoptosis) by acting as sponges of miR-324-5p and miR-361-3p to promote GBM progression (31). CircRNAs and corresponding signaling pathways in glioma are summarized in **Table 1**.

**Table 1:** CircRNAs and corresponding signaling pathways in glioma.

CircRNA (id in circBase)	Genomic position	Gene symbol	CircRNA type	Regulation	Target	Downstream	Pathway function	Clinical significance	References
hsa_circ_0005198	chr13:25072253-25077915	PARP4	exonic	Up	miR-198	TRIM14	Proliferation, apoptosis	TMZ resistance	(32)
hsa_circ_0002330	chr8:131172109-131193126	ASAP1	exonic	Up	miR-502-5p	NRAS	Proliferation	TMZ resistance	(33)
hsa_circ_0072083	chr5:32354455-32444434	ZFR	exonic	Up	miR-1252-5p	NANGO	Proliferation, migration, invasion	TMZ resistance	(34)
hsa_circ_0110757	chr1:150549927-150550695	MCL1	exonic	Up	miR-1298-5p	ITGA1	Invasion	TMZ resistance	(35)
hsa_circ_0042003	chr17:8134593-8141947	CTC1	exonic	Up	NA	NA	Proliferation, apoptosis	TMZ resistance	(36)
hsa_circ_0005198	chr13:25072253-25077915	PARP4	exonic	Up	miR-1294	NA	Proliferation, apoptosis, metastasis	Prognosis	(37)
hsa_circ_0082374	chr7:129948146-129964020	CPA4	exonic	Up	miR-760	MEF2D	Proliferation, migration, invasion, apoptosis	Radioresistance	(38)
hsa_circ_0073237	chr5:82832825-82838087	VCAN	exonic	Up	miR-1183	NA	Proliferation, migration, invasion	Radioresistance	(39)
hsa_circ_0074026	chr5:134363423-134365011	PITX1	NA	Up	miR-329-3p	NEK2	Glycolysis	Radioresistance	(40)
hsa_circ_0098619	chr12:46325288-46328288	SCAF11	exonic	Up	miR-145-5p	NA	Proliferation, migration, invasion	Ropivacaine therapy	(41)
hsa_circ_0082846	chr7:148543588-148543690	EZH2	exonic	Up	miR-181b-5p	NA	Proliferation, migration, invasion	Lidocaine therapy	(42)
hsa_circ_0002755	chr7:73770739-73771807	CLIP2	exonic	Up	miR-628-5p	MAGT1	Proliferation, migration, invasion, apoptosis, glycolysis	Sevoflurane therapy	(43)
hsa_circ_0047688	chr18:48189458-48190874	MAPK4	exonic	Up	miR-125a-3p	P38/MAPK	Proliferation, apoptosis	Pathological stage, prognosis	(44)
hsa_circ_0034642	chr15:41191341-41196173	VPS18	exonic	Up	miR-1205	BATF3	Proliferation, migration, invasion	NA	(29)
hsa_circ_0001946	chrX:139865339-139866824	CDR1	exonic	Up	miR-671-5p	CDR1	Proliferation, migration, invasion	NA	(45)
hsa_circ_0076248	chr6:37787306-38084515	ZFAND3	exonic	Up	miR-181a	SIRT1	Proliferation, migration, invasion	NA	(46)
hsa_circ_0000284	chr11:33307958-33309057	HIPK3	exonic	Up	miR-124-3p	STAT3	Proliferation, migration, invasion	NA	(47)
hsa_circ_0000284	chr11:33307958-33309057	HIPK3	exonic	Up	miR-654	IGF2BP3	Proliferation, migration, invasion	NA	(48)
hsa_circ_0000284	chr11:33307958-33309057	HIPK3	exonic	Up	miR-421	ZIC5	Proliferation, invasion, apoptosis	TMZ resistance	(49)
hsa_circ_0000284	chr11:33307958-33309057	HIPK3	exonic	Up	miR-524-5p	KIF2A/P13K/AKT	Proliferation, migration	TMZ resistance	(50)
hsa_circ_0000284	chr11:33307958-33309057	HIPK3	exonic	Up	miR-124	CCND2	Proliferation, migration, invasion	NA	(51)
hsa_circ_0081519	chr7:100400186-100417918	EPHB4	exonic	Up	miR-637	SOX10/Nestin	Stemness, proliferation and glycolysis	Prognosis	(52)
hsa_circ_0027068	chr12:57031958-57033091	ATP5B	exonic	Up	miR-185-5p	HOXB5/JAK2/STAT3	Proliferation	Prognosis	(53)
hsa_circ_0033009	chr14:93180720-93207524	LGMN	exonic	Up	miR-127-3p	LGMN	Proliferation, invasion	Prognosis	(54)
hsa_circ_0001103	chr2:224862831-224866639	SERPINE2	NA	Up	miR-361-3p/miR-324-5p	BCL2	Proliferation	Diagnosis, prognosis	(31)
hsa_circ_0090956	chrX:69606467-69607147	KIF4A	exonic	Up	miR-139-3p	Wnt/β-catenin	Proliferation, migration and invasion	NA	(55)
hsa_circ_0030018	chr13:38136718–38161065	POSTN	NA	Up	miR-361-5P	TPX_2/_AKT	Proliferation, apoptosis and aerobic glycolysis	NA	(30)
hsa_circ_0030018	chr13:38136718–38161065	POSTN	NA	Up	miR-1205	NA	Proliferation, migration and invasion	Tumor size, WHO grade, prognosis	(56)
hsa_circ_0001730	chr7:100410368-100410830	EPHB4	exonic	Up	miR-326	Wnt/β-catenin	Proliferation, migration, invasion	NA	(57)
hsa_circ_0001801	chr8:52773404-52773806	PCMTD1	exonic	Up	miR-224-5p	mTOR	Proliferation, migration, invasion	NA	(58)
hsa_circ_0001742	chr7:128845043-128846428	SMO	exonic	Up	miR-338-3p	SMO	proliferation, migration, invasion	NA	(59)
hsa_circ_0082374	chr7:129948146-129964020	CPA4	exonic	Up	let-7	CPA4	Proliferation, migration, invasion	Prognosis	(60)
hsa_circ_0005660	chr19:13135834-13136366	NFIX	exonic	Up	miR-34a-5p	Notch1	Proliferation, migration, invasion, apoptosis	NA	(61)
hsa_circ_0049658	chr19:13183860-13192669	NFIX	exonic	Up	miR-378e	RPN2	Proliferation, migration, invasion	Prognosis	(62)
hsa_circ_0006404	chr6:108984657-108986092	FOXO3	exonic	Up	miR-138-5pNAmiR-432-5p	NFAT5	Proliferation, migration, invasion,	NA	(63)
hsa_circ_0001162	chr20:44643022-44645125	MMP9	NA	Up	miR-124	CDK4/AURKA	Proliferation, migration, invasion, EMT	NA	(64)
hsa_circ_0088732	chr9:130914461-130915734	LCN2	exonic	Up	miR-661	RAB3D	Proliferation, migration, invasion, EMT	NA	(65)
hsa_circ_0005460	chr9:36581640-36589649	MELK	exonic	Up	miR-593	EphB2	Proliferation, migration, invasion, EMT	Therapeutic strategy	(66)
hsa_circ_0003949	chr7:136935976-136939721	PTN	exonic	Up	miR-145-5pNA miR-330-5p	SOX9/ITGA5	Proliferation, self-renewal	NA	(67)
hsa_circ_0000876	chr19:4405908-4409756	CHAF1A	exonic	Up	miR-211-5p	HOXC8	Proliferation	Prognosis, diagnosis	(68)
hsa_circ_0074027	chr5:134363423‐134369964	PITX1	NA	Up	miR-518a-5p	IL17RD	Proliferation, migration, invasion	NA	(69)
hsa_circ_0067934	chr3:170013698-170015181	PRKCI	exonic	Up	miR-545	NA	Proliferation, migration, invasion	NA	(70)
hsa_circ_0016767	chr1:228285042-228286913	ARF1	exonic	Up	miR-342-3P	ISL2	Angiogenesis	Prognosis	(71)
hsa_circ_0026782	chr12:56094682-56094938	ITGA7	exonic	Up	miR-34a-5p	VEGFA	Proliferation, migration, invasion, angiogenesis	Screening, diagnosis, therapeutic strategy	(72)
hsa_circ_0098619	chr12:46325288-46328288	SCAF11	exonic	Up	miR-421	SP1/VEGFA	Proliferation, migration, invasion	Prognosis	(73)
hsa_circ_0075686	chr6:16299342-16328701	ATXN1	NA	Up	miR-526b-3p	MMP2/VEGFA	Migration, angiogenesis	NA	(74)
hsa_circ_0000005	chr1:1586822-1650894	CDK11A	exonic	Up	miR-138-5p	SOX13	Angiogenesis	NA	(75)
hsa_circ_0008278	chr2:120885263-120932580	EPB41L5	exonic	Down	miR-19a	EPB41L5p-AKT	Inhibit proliferation, migration and invasion	Prognosis	(76)
hsa_circ_0001141	chr20:33001547-33037285	ITCH	exonic	Down	miR-214	Wnt/β-catenin	Inhibit proliferation, migration and invasion	NA	(77)
hsa_circ_0099761	chr13:101997616-102051516	NALCN	exonic	Down	miR-493-3p	PTEN	Proliferation	Prognosis, therapeutic target	(78)
hsa_circ_0001417	chr4:73950965-73958017	ANKRD17	exonic	Up	miR-195-5p	ETV1	BTB permeability	NA	(79)
hsa_circ_0005684	chr9:19286766-19305525	DENND4C	exonic	Up	miR-577	ZO-1/occluding/claudin-1	BTB permeability	NA	(80)
hsa_circ_0000080	chr1:62910408-62914337	USP1	exonic	Up	miR-194-5p	FLI1	BTB permeability	NA	(81)
hsa_circ_0014130	chr1:151206672-151212515	PIP5K1A	exonic	Up	miR-515-5p	TCF12/PI3K/AKT	Proliferation, migration, invasion, EMT	Tumor size, WHO grade	(82)
hsa_circ_0061868	chr21:44513065-44527688	U2AF1	NA	Up	miR-7-5p	NOVA2	Proliferation, apoptosis	Prognosis	(83)
hsa_circ_0077232	chr6:86180954-86205509	NT5E	exonic	Up	miR-422a	NA	Proliferation	NA	(84)
hsa_circ_0002142	chr7:140301202-140302342	DENND2A	exonic	Up	miR-625-5p	HIF1α	Migration, invasion	NA	(85)

NA, not available; TMZ, temozolomide; EMT, the epithelial mesenchymal transition; BTB, Blood-tumor barrier.

### 2.4 Interactions With Proteins

RNA-binding proteins (RBPs) are proteins that bind to double- or single- stranded RNA. RBPs in glioma are involved in different biological processes including RNA splicing, processing, localization and transport (**Figure 2C**). For example, HNRNPA2B1 could enhance the expression of the oncogenic isoforms of many tumor suppressor genes (e.g., *RON*, *BIN1*, *WWOX*, and *c-FLIP*) in glioma by modulating the splicing of these genes, thereby promoting glioma progression and aggressiveness (86). Liu et al. found that splicing factor *SRSF10* can bind to *Alu* elements on both sides of the circ-ATXN1 pre-mRNA (74), thus promoting the generation of circ-ATXN1 and promoting the proliferation, migration and tube-forming capacity of GECs (glioma-exposed endothelial cells, GECs) *via* circ-ATXN1/miR-526b-3p/MMP2/VEGFA pathway. Matrix metalloproteinases (MMPs; e.g., MMP2) and vascular endothelial growth factor (VEGF) are involved in malignant tumor, inflammation and tissue remodeling (87). Recently, He et al. (75) demonstrated that FUS binds to circ_002136, which positively regulates the SOX13 transcription factor by sponging miR-138-5p. Interestingly, SOX13 cannot only promote SPON2 expression through combining the SPON2 promoter region but also activates the FUS promoter to form a feedback loop that promotes the viability and antigenic capacity of GECs. In contrast, knockout of FUS and circ_002136 can reduce tube-forming ability of GECs. In addition, Moloney leukemia virus 10 (MOV10), belongs to the RNA helicase superfamily, can bind to circ-DICER1 and regulate glioma angiogenesis through the circ-DICER/miR-103a-3p (miR-382-5p)/ZIC4 pathway (88). Moreover, RBPs may modulate glioma progression through specific metabolism pathways (carbon metabolism and amino acid metabolism). In addition, a recent study demonstrated that the abundance of RBPs increases with the glioma grade (89).

In addition to the above mentioned RBPs, circRNA could bind to proteins to form a specific circRNA-protein complex (circRNP), which exerts its function in the regulation of the subcellular localization of proteins and their post-transcriptional levels, thus further affecting the cell cycle, angiogenesis and tumor processes. For instance, circFOXO3 mediates the post-translational modifications of proteins, including ubiquitination and phosphorylation. Du et al. found that circFOXO3 can interact with proteins to form a ternary complex (circFOXO3-MDM2-p53) and then promote MDM2-induced p53 ubiquitin degradation to prevent the ubiquitination of FOXO3 and increasing its synthesis in breast cancer cell lines (90). Similarity, using pull-down assays, they also found that circFOXO3 regulates the cell cycle in the form of the circFOXO3-p21-CDK2 ternary complex in mouse non-cancer cells, which can enhance the interplay of CDK2 with p21 and inhibit the phosphorylation of CDK2. These effects ultimately attenuate the cell cycle at the G1 phase by blocking the G1/S transition and S phase processes (91). Notably, circRNA can disrupt protein-protein interactions. Circ-CCNB1 interacts with two key mitosis-related proteins to form a ternary circ-CCNB1-cyclinB1-CDK1 complex in malignant glioma cell lines. This complex can abolish the role of CCNB1 in enhancing cell migration, invasion and proliferation to produce anti-tumor effect (92). However, CDK1 and CCNB1 are highly expressed in glioblastoma and their levels significantly correlate with poor survival (93). Therefore, circ-CCNB1 could potentially be used against glioma as a treatment, further research is needed to explore this mechanism. Furthermore, circRNA can bind to a single protein to affect its function. Recently, a study constructed rabies virus glycoprotein-circSCMH1 EV and studied them in acute ischemic stroke models in mice and monkeys. They found that circSCMH1 interfered with the inhibitory function of transcription factor MeCP2 (methyl-CpG binding protein 2), thereby upregulating its downstream genes to promote brain function and neuroplasticity and inhibiting peripheral immune cell infiltration and glial cell activation (94). This study also demonstrated that circRNA could be engineered into circRNA-EVs for administration as a promising therapeutic strategy.

Overall, existing studies have shown that there may be several roles for circRNA-protein interactions: circRNAs alter gene expression by binding to *cis* elements to regulate transcription factors or epigenetics; CircRNAs form a protein-circRNA-mRNA ternary complex to regulate tumor genesis. The interactions of circRNAs with proteins has greatly enriched their function.

#### 2.4.1 Regulation of Transcription and Splicing

It is speculated that nuclear circRNAs may be involved in the transcription and splicing process according to the published studies. CiRNA and EIciRNA predominantly localize in the nucleus and affect parental gene expression (95, 96) (**Figure 2A**). For example, ci-ankrd52 is abundant at the transcriptional loci of its parent gene (ANKRD52) and plays a *cis*-regulatory role on the transcriptional levels by positively modulating the pol II complex (96). However, the transcription-enhancing effects of EIciRNAs (e.g., circEIF3J and circPAIP2) are mediated by the formation of EIciRNA–U1 snRNP complexes, which may further interact with the Pol II transcription complex (95).

#### 2.4.2 Role in Translation

Cap-dependent mechanism is the main translation method in eukaryotes and circRNA was once considered incapable of encoding proteins due to lack of a 5’-end cap and a 3’-end poly (A) tail. However, researchers have recently found that some circRNAs harbor open reading frame (ORF) structures in their sequences and can be translated into proteins *via* internal ribosome entry site (IRES)-driven initiation mechanism, in which the 40S ribosome can directly bind to IRES elements without scanning from the 5′ end of mRNAs (97). In addition, *ITAFs* could interplay with IRES elements and thus initiate IRES-driven translation by recruiting ribosomes to the IRES (98) (**Figure 2D**). Chen et al. found that 18S rRNA complementarity and a distinct secondary structure (SuRE) on the IRES are important for driving circRNA translation (99). Moreover, circRNAs may also encode proteins through alternative mechanism driven by the m6A modification (100). This kind of modification preferentially appears in the long exon regions of circRNAs and is enriched around the upstream and middle exon regions (101). CircRNAs could be methylated by m6A with the help of the METL3/METTL14 complex (100) and circRNAs with m6A-induced ribosome engagement sites (MIRES) could initiate translation process by recruiting YTHDF3 (reader) that can recruit other translation initiation factors, including eIF4G2 (102). Intriguingly, circRNA-encoded proteins may serve as a hit in the stress response, because MIRESs could directly bind to the initiation factor *eIF3* to facilitate translation when cells suffer from various unfavorable environmental stresses (e.g., heat shock, chemicals, or hypoxia) (103–105). Notably, circRNA could also translate proteins through rolling circle amplification (RCA) mechanism. To be specifically, the number of nucleotide of these circRNAs is multiple of three and they only have start codons but lack of stop codons, which theoretically means high-molecular weight proteins could be produced once translation begins (106) (**Figure 2D**).

A growing number of studies have shown that circRNAs which proven to be translatable in glioma may play a role in tumor progression. For example, circ-FBXW7 is downregulated in glioma and a cross‐linked ORF in circ-FBXW7, which is formed by the covalent connection of exon 3 and exon 4 of the *FBXW7* gene, encodes a novel 21-kDa protein called FBXW7-185aa in an IRES-driven manner (107). Moreover, FBXW7‐185aa can interact with the deubiquitinating enzyme USP28 to protect USP28 from interacting with FBXW7α, resulting in shorter half‐life of c‐Myc and inhibiting malignant glioma progression. Additionally, Zhang et al. (108) demonstrated that an ORF in circ-SHPRH can encode a functional protein (SHPRH-146aa) in an IRES-driven way. It is worth noting that the translation process of circ-SHPRH has overlapping initiation and termination codons, in which the start and stop codons share an A base (‘UGAUGA’). Full-length SHPRH is an E3 ligase that can specifically mediate the ubiquitination-mediated degradation of proliferating cell nuclear antigen (PCNA) (109). SHPRH-146aa overexpression can also induce PCNA degradation, which can be inhibited by the proteasome inhibitor MG132. SHPRH-146aa may increase SHPRH level by extending its half-life and protecting full-length SHPRH from denticleless E3 ubiquitin protein ligase (DTL)-mediated degradation, thereby promoting PCNA degradation and inhibiting cell growth and tumorigenesis. In addition, patients with high expression of SHPRH-146aa might have a better prognosis. Researchers had identified an 87-aa peptide encoded by the short open reading frames (sORF) in circPINTexon2, which was cyclized from exon 2 of LINC-PINT (110). sORF dependent translation initiation is one of the translation ways. Both m6A dependent and IRES dependent translation initiation are belonging to circRNA translation initiation. Notably, downregulation of PINT87aa expression in glioma tissues correlates with poor prognosis and PINT-87aa overexpression can trigger G1-phase cell cycle arrest and inhibit neurosphere-forming capabilities. Moreover, PINT87aa can potentially bind to the polymerase-associated factor (PAF1) complex to suppress oncogenic transcriptional elongation, resulting in the inhibition of glioblastoma growth. Zhang et al. have also demonstrated a novel protein (AKT3-174aa) that is encoded by circAKT3 and downregulated in glioma tissues. This protein can interact with p-PDK1 and act as a tumor suppressor by preventing AKT Thr-308 phosphorylation and inhibiting the malignant phenotype and glioma progression by decreasing the RTK/PI3K/AKT signaling pathway (111, 112). Notably, a recent study demonstrated that the tumor suppressor gene *E-cadherin* could be cyclized to form circ-E-Cad in an IRES-driven manner, and circ-E-Cad could encode a 254-amino-acid protein called circRNA-encoded E-cadherin (C-E-Cad), this protein activates the epidermal growth factor receptor (EGFR) in an autocrine/paracrine fashion. Interestingly, researchers also found that C-E-CAD targeting therapy could significantly inhibit GBM process and prolong the survival of nude mice (113). Liu et al. recently found that circ-EGFR could translate a new protein called rolling translated EGFR (rtEGFR) through RCA mechanism, this protein could reduce the endocytosis and degradation of EGFR (114).

In conclusion, peptides and proteins (e.g., SHPRH-146aa, FBXW7-185aa, PINT-87aa, AKT3-174aa, circ-E-cad, rtEGFR) that encoded by circRNAs play an important role in glioma progression by modulating metabolic reprogramming, the epithelial mesenchymal transition (EMT) transition and the stability of oncogenic proteins (e.g., c-Myc). These peptides and proteins might turn out to be novel clinical biomarkers and therapeutic targets. The specific translational mechanisms for circRNA remain unclear. However, studies have shown that other short sequences may also have the ability to drive translation of circRNA effectively (e.g., methylation of adenosine) (115).

#### 2.4.3 Extracellular Vesicles circRNAs in Glioma

Extracellular vesicles (EVs) are cell-derived membranous vesicles that could be secreted by all cell types (116). EVs can carry a variety of bioactive molecules to regulate multiple functions of recipient cells. As critical mediators of intercellular communication, EVs are implicated in the occurrence and pathogenesis of various ailments, including tumors (117, 118).

Recently, circRNAs have been detected among the bioactive compositions of glioma extracellular vesicles (**Figure 2E**). Thus, EVs enable circRNAs to enter circulation and interact with recipient cells to carry out multiple biological functions (119). Glioma-derived EVs circRNAs may have implications for the radio-sensitivity of the individuals, thereby affecting treatment efficiency. For example, Zhao et al. (120) found that circRNA-ATP8B4 that from radio-resistant glioblastoma multiforme (GBM)-derived EVs sponged miR-766 to facilitate glioma cell radioresistance. Besides, glioma derived EVs circRNAs might also have the ability to induce endothelial cell angiogenesis (121). Furthermore, researchers found that miR-21 levels (associated with malignant relapse and spinal/ventricular metastasis) in cerebrospinal fluid (CSF) derived EVs from GBM patients was significantly higher than that of controls, while there was no difference in serum-derived EVs miR-21 expression (122, 123). These studies suggested that CSF-derived EVs circRNAs could be used as promising biomarkers for glioma diagnosis and prognosis. Additionally, circRNA can be selectively packaged into EVs and the phospholipid bilayer membrane structure of EVs has a protective effect on circRNA because of its resistance to ribonuclease degradation. Additionally, EVs have good biocompatibility that can overcome the blood-brain barrier (BBB), and Lai et al. developed a EVs reporter system that can monitor the EVs biodistribution over time *in vivo* imaging, they found that systemic injection of EVs reaches the tumor sites within an hour (124, 125). Therefore, EVs circRNAs hold great potential for clinical diagnosis and treatment of glioma. However, it should be noted that the exosome may be a double-edged sword for circRNA because it can reduce circRNA accumulation. Lasda et al. pointed out that cells excrete circRNAs with the help of extracellular vesicles and these circRNA-containing vesicles can be absorbed and cleared by specific cells, including macrophages (119).

In conclusion, EVs circRNAs hold great potential for clinical diagnosis and treatment of glioma but still in its infancy, whether the process of relocating circRNA from the cytoplasm to the EVs is by active transfer or passive inclusion remains to be studied. Besides, a precondition of EVs circRNA could be designed as therapeutic factors delivery is that EVs locate cargos to the target site accurately. However, the underlying mechanisms that EVs target the specific cell type remains largely unknown. Additional research is needed to explore this mechanism.

## 3 Expression and Significance

Glioma is the most prevalent malignant brain tumor. Many ncRNAs are responsible for the biogenesis and progression of glioma. CircRNA is a newly identified RNA involved in many diseases, including glioma.

### 3.1 In the Central Nervous System

Many researchers have demonstrated that circRNA showed higher expression in mammalian brain tissue than other tissues (126). More specifically, the expression of these circRNAs is different between different anatomical structures of the brain (e.g., striatum, olfactory cortex, prefrontal cortex and hippocampus) (126). CircRNA expression is positively correlated with synaptic structure because genes related to synapses are more likely to be circularized (127). Interestingly, many circRNAs change their abundance abruptly during synaptogenesis (128), which indicates that brain-enriched circRNAs are dynamically modulated during neuronal maturation and development. The loss of a mammalian circRNA locus may lead to miRNA imbalance, thus affecting brain function (129). CircHomer1 modulates certain structural changes in synapses during neuronal plasticity and development (128). Additionally, Westholm et al. (130) found that in drosophila, the formation of circRNA may tend to reside in the surrounding of the cerebral long intron regions and accumulate in the aging brain tissue.

Notably, the circRNA-miRNA-mRNA network plays a key regulatory role in the neuropathological mechanisms. Take CDR1as (ciRS-7) as an example, it is abundant in the human brain, especially in excitatory neurons and can serve as a miRNA-7 sponge, however, miRNA-7 is a modulator of ubiquitin protein ligase A (UBE2A) and α-synuclein (α-Syn). Hence, ciRS-7 is involved in the progression of chronic neurodegenerative diseases (e.g., Alzheimer’s disease and Parkinson’s disease) (131, 132). CircRNA was found to participate in secondary brain injury after an acute central nervous system injury (e.g., stroke and neonatal hypoxic-ischemia). Moreover, one study revealed underlying connections between depression or moyamoya disease and circRNA expression (127, 133).

### 3.2 Expression in Glioma

A total of 46 cases of glioma and normal brain tissues were analyzed by microarray. Results showed that there were differences in the expression of 476 circRNAs (a total of 572 detected circRNA) between normal and tumor tissues (134). Moreover, 468 circRNAs were highly expressed in normal tissues compared to GBM tissues. The specific mechanism of these circRNAs in the pathogenesis of gliomas remains to be elucidated. Xu et al. (61) used circRNA analysis tools to analyze three pairs of RNA-seq-related data for glioma and normal brain tissue. Twelve differentially expressed circRNAs were screened, of which circNFIX was the only overexpressed circRNA in glioma. Similar studies by other labs revealed that most differentially expressed circRNAs in GBM were downregulated (135, 136).

In short, most studies focused on those differentially expressed circRNAs in gliomas. We should note that the expression of circRNAs may have a high degree of specificity and variability among glioma patients. The exact expression pattern of circRNA needs further validation with larger sample sizes. Meanwhile, differences in methods (microarray *vs* RNA-Seq) may also play a role. Total RNA-Seq is less sensitive than circRNA microarrays (137). In addition, different sample sizes may cause mutations in DEGs (difference expression genes). Hundreds of circular RNAs are abundant in mammalian brains (128), circRNAs are closely related to the pathophysiological process of brain tumors (127). To find commonly regulated circRNAs (hub genes) is very important and requires further investigation.

### 3.3 Roles in Diagnosis and Prognosis

CircRNAs could be detected in blood, secretions and tissues and their unique characteristics (e.g., high expression, stability and temporal and spatial specificity) imply that circRNAs may have the ability to detect tumors (138). According to published studies, there are several types of circRNAs associated with the prognosis of glioma. The first type includes circRNAs that can encode proteins, such as circ-SHPRH, circ-FBXW7 and circAKT3. These circRNAs, which can encode tumor suppressor proteins, are underexpressed in glioma and associated with poor prognosis. The second type consists of exosomal circRNAs. CircRNAs are widely abundant and stable in exosomes (139) and serve a role in drug resistance and the delivery of targeted drug molecules. Some other circRNAs (e.g., circEPHB4, circCPA4, circ-MAPK4, circ-POSTN, circNFIX, circSCAF11, circ-U2AF1 and circLGMN are also associated with poor patient prognosis in glioma (44, 52, 54, 56, 60, 62, 73, 83). Circ-MAPK4 and circ-POSTN were also associated with improved tumor size and WHO grade. Additionally, circPIP5K1A ectopic expression may also be associated with glioma volume and histopathological grade (82).

Further investigation is needed to identify whether circRNAs could be considered novel biomarkers for glioma prognosis. Notably, a previous study reported that a novel carcinogenic circRNA derived from reverse splicing of the non-small cell lung cancer fusion gene *EML4-ALK* variant 3b (V3b) could be detected in the plasma of patients carrying the *EML4-ALK* gene, indicating that circRNA has potential in cancer diagnosis and clinical treatment as a liquid biopsy marker (140). Relations between circRNAs and the occurrence and development of glioma still needs further exploration.

## 4 Mechanisms in Glioma Progression

### 4.1 Glioma Genesis and Proliferation

CircEPHB4 is upregulated in glioma and increases SOX10 and Nestin expression levels by competitively binding to miR-637, which ultimately stimulates tumor cell stemness and self-renewal (52). The multi-lineage differentiation and self-renewal capacities of glioma stem cells (GSCs) contribute to tumor proliferation and recurrence. Notably, recent studies have found that circATP5B and circCHAF1A were upregulated in glioma and GSCs, promoted GSCs proliferation through miR-185-5p/HOXB5 and miR-211-5p/HOXC8 axis, respectively (53, 68). In addition, circRNA can mediate the genesis and proliferation of glioma cells through cell cycle regulation and apoptosis inhibition. In addition to FBXW7-185aa described earlier, circ-MAPK4 sponges miR-125a-3p to inhibit glioma cell apoptosis *via* downregulating P38/MAPK phosphorylation levels (44). Activation of P38/MAPK is correlated with the apoptosis of nerve cells and tumor cells (82, 83).

### 4.2 Glioma Cell Migration and Invasion

CircRNAs acting as a miRNA sponge and forming the circRNA-miRNA-mRNA axis is an important mechanism of glioma invasion and metastasis. In addition to binding to an RNA binding protein, circFOXO3 can also serve as competitive endogenous RNA (ceRNA) to upregulate NFAT5 by sponging miR-138-5p and miR-432-5p and enhance GBM cell proliferation and invasion ability (63). Additionally, circSMO742 contributes to cell proliferation and invasion by targeting miR-338-3p and upregulating smoothened (SMO) expression levels in glioma tissues (59). SMO not only enhances invasion of tumor cells but also regulates gene expression by transferring GLI1 from the cytoplasm to the nucleus, thereby improving the stemness of cancer stem cells and inhibiting apoptosis (141). Moreover, circCPA4, circPITX1, circPRKCI, circ_001350 and circHECTD1 are significantly upregulated in glioma and accelerate glioma cell aggressiveness *via* the let7/CPA4, miR-518a-5p/IL17RD, miR-545, miR-1236 and miR-296-3p axes (60, 69, 70, 142, 143). Noteworthy, circHIPK3, which is highly expressed in glioma and correlated with poor prognosis, can function as miRNA sponges for multiple miRNAs, including miR-124-3p, miR-654 and miR-124, to elevate expression levels of *STAT3, IGF2BP3* and *CCND2*, respectively, leading to increased cell invasiveness and metastasis (47, 48, 51). In addition, circHIPK3 is present in exosomes secreted by glioma cells. Exosomal circHIPK3 promotes cancer development and temozolomide (TMZ) resistance by controlling the miR-421/ZIC5 axis (49). A recent study showed that circNALCN was downregulated in glioma and inhibited tumorigenesis and invasion through targeting miR-493 (78).

Aberrantly expressed circRNAs in glioma may also play essential roles in tumorigenesis through cancer signaling pathways. Circ_0001730 functioned as miR-326 sponges to positively modulate the Wnt/β-catenin pathway, which can induce tumor cell invasion and migration and the EMT process in glioblastoma cells (57). Many epidermal growth factors and their corresponding receptors are implicated in EMT. CircZNF292 can interact with other related genes, including cyclin A, VEGFR and EGFR, to promote tumorigenesis and invasion. Silencing circZNF292 can block cell cycle in the S/G2/M phase, inhibiting glioma cell migration and tube formation (144). Moreover, circKIF4A is also conspicuously upregulated in glioma tissues and exerts its tumor-promoting role *via* activating the Wnt/β-catenin pathway by sponging miR-139-3p (55). Activation of PI3K/AKT signaling is common in most cancers, including glioma. The natural product matrine can induce apoptosis and autophagy in glioma cells by downregulating the PI3K/AKT and Wnt-β-catenin pathways (145). CircPIP5K1A and circNT5E can modulate PI3K/AKT downstream signaling *via* sponging miR-515-5p and miR-422a, respectively, thereby promoting glioma proliferation and metastasis (82, 84). Furthermore, circPCMTD1 and hsa_circ_0037251 promote glioma cell proliferation and metastasis by regulating the mammalian target of rapamycin (mTOR) pathway *via* sponging of miR-224-5p and miR-1229-3p, respectively (146). mTOR is a conserved serine/threonine protein kinase that coordinates cellular growth and metabolism through interactions with various proteins. When this process goes wrong, mTOR reprograms normal cells to divide abnormally and sends signals that encourage tumor cells to develop, grow, metastasize and invade healthy tissue (58, 147). Remarkably, the same circRNA may mediate tumor invasion and metastasis through multiple signaling pathways. For example, the highly expressed circNfix in glioma can act through the circNfix/miR-378e/RPN2 axis (62) or be a sponge for miR-34a-5p and upregulate the target gene *NOTCH1* to enhance glioma invasion through the Notch signaling pathway (61). CircSMARCA5 is dramatically downregulated in GBM and acts as tumor suppressor by tethering serine/arginine-rich splicing factor 1 (*SRSF1*). *SRSF1* is a positive controller of cell migration and adhesion by upregulating poly pyrimidine tract binding protein 1 (PTBP1) (148).

### 4.3 Epithelial-to-Mesenchymal Transition

EMT is a process by which cells lose their polarity and acquire the ability to migrate, invade and metastasize (149). A study conducted by Wang et al. (64) supported that eIF4A3-induced circMMP9 acts as miR-124 sponges to upregulate CDK4 and AURKA, promoting GBM cell proliferation and invasion. Interestingly, the circMMP9/miR-124 axis regulates the expression of EMT markers (i.e., E-cadherin, snail and vimentin) in GBM cells. In addition, hsa_circ_0088732 derived from the cyclization of Lcn2, accelerates glioma progression, migration, invasion and EMT by affecting the miR-661/RAB3D axis (65). CircPTN is an oncogenic factor. Its overexpression can rescue the inhibition regulation of the oncogenic proteins SOX9 and ITGA5 by miR-145-5p and miR-330-5p in GSCs, respectively, resulting in increased proliferation, self-renewal and stemness of GSCs (67). Simliar to circPTN, circMELK and circPARP4 regulate GBM EMT and stemness of GSCs through upregulating oncogenic protein EphB2 and FUT4 by acting as sponges for miR-593 and miR-125a-5p, respectively (66, 150).

Overall, circRNAs can modulate the cell cycle and apoptosis by regulating the classic circRNA-miRNA-mRNA axis and participating in tumor-related signaling pathways, thereby enhancing glioma cell growth, invasion and metastasis. Exploring the cancer-promoting mechanisms of circRNA in glioma is of great value for understanding molecular biology of gliomas and developing new targeted therapies.

### 4.4 Mechanisms in Glioma Microenvironment

There is growing evidence that communications and interactions between circRNAs and significant components in tumor microenvironment (TME) influence tumor initiation, progression and metastasis. The TME harbors two main components, cellular and non-cellular secreted components. The former comprise infiltrating immune cells, inflammatory cells, cancer-associated endothelial cells (CAES), cancer-associated fibroblasts (CAFs) and cancer stem cells (CSCs). The latter include cytokines, growth factors, metabolites and the extracellular matrix (ECM) (151). Metabolism includes glucose metabolism, lipid metabolism, amino acid metabolism and other metabolic regulatory mechanisms. Among these, glucose metabolism changes are highly correlated with the glioma genesis and development. Cancer and stromal cells usually lead to nutrient and oxygen limitation, thus establishing anoxic microenvironments. Hyperosmotic inducible factor 1 α (HIF1α) is a hypoxia marker that greatly influences malignant transformation and tumor metastasis (152). It can promote tumor angiogenesis and glucose metabolism, thus affecting tumor cell proliferation (153). In addition, hypoxia can induce EMT and invasion by regulating EMT-associated transcriptional factors, including TWIST, SNAIL, ZEB1 and ZEB2 (154). High HIF1α expression levels in surgically resected glioma tissue are significantly correlated with shorter overall survival (155). CircDENND2A derived from the *DENND2A* gene is highly expressed in HIF1α-associated glioma cells and facilitates tumor cell aggressiveness by competitive binding to miR-625-5p (85).

#### 4.4.1 Roles in Glioma Angiogenesis

CAEs that cover the inner surface of tumor vasculatures and lymphatics could promote tumor angiogenesis (156). CAFs are those fibroblasts that remain in the prolonged activated state, which exert their role in tumorigenesis and metastasis *via* secreted growth factors, such as fibroblast growth factor (FGF) (157). Recently, Zou et al. (157) utilized online analytical tools to evaluate relations between CDR1as expression and TME. Results showed that high expression of CDR1as was correlated with CAEs and CAFs infiltration. In addition, CDR1as might upregulate the TGF-β pathway to interact with the ECM receptor, thus exerting an influence on tumor-associated vasculature formation, tumor tissue immunity and stromal cell infiltration. Moreover, circSCAF11 and circITGA7 can promote glioma angiogenesis and tumor occurrence through miR-421/SP1/VEGF and miR-34a-5p/VEGFA axis, respectively (72, 73). Furthermore, Jiang et al. (71) recently discovered that circRNA ARF1 (cARF1) upregulates ISL2 by sponging miR-342-3p in GSCs. ISL2 facilitates the angiogenesis, proliferation and invasiveness of human brain microvessel endothelial cells (hBMECs) *via* VEGFA-mediated ERK signaling. Interestingly, U2AF2, which is upregulated by ISL2, can bind to cARF1 and promotes its stability and expression, forming a feedback loop in GSCs. Moreover, SRSF1 could induce the aberrant splicing of VEGFA pre-mRNA and thus leading to an increased proportion of pro-angiogenic and anti-angiogenic subtypes (Iso8a/Iso8b) (158).

Several other angiogenic factors can also directly or indirectly affect CAE-mediated angiogenesis. For example, overexpressed circ-DICER1 in GECs can act as sponges for miR-103a-3p and miR-382-5p, induces the upregulation of the downstream target Hsp90β by weakening the inhibitory effect on the ZIC family member 4 (ZIC4) and activates the PI3K/AKT pathway to mediate GEC angiogenesis (88). Additionally, circ-SHKBP1 upregulates the FOXP1/FOXP2 pathway by targeting miR-544a/miR-379. Interestingly, FOXP1/FOXP2 can increase the expression of pro-angiogenic factors, which, in turn, promotes GEC viability and angiogenesis *via* PI3K/AKT and ERK1/2 pathways (159).

#### 4.4.2 CircRNA and Glucose Metabolism

Aerobic glycolysis, also known as the “Warburg effect,” refers to the catabolism process in which tumor cells consume glucose and produce a large amount of lactic acid even when oxygen supply is sufficient (160). Compared to normal brain, glioma is characterized by increased aerobic glycolysis, leading to hypoxic local tissue, the production of HIF-1α and TGF-β, the activation of immunosuppressive CD4^+^ T cells and inhibition of NK cell activity, creating an acidic, hypoxic and immunosuppressive TME that is conducive to malignant invasion, metastasis and immune resistance (161–163).

Emerging studies revealed that miRNA might modulate glycolysis by manipulating the expression and activity of glycolytic transporters and rate-controlling enzymes, including hexokinase (HK), 6-phosphate fructokinase (PFK) and pyruvate kinase (PK) (164). Because circRNA may act as a sponge for miRNA, it is speculated that circRNA may indirectly participate in tumor metabolism. In addition, the possibility that circRNA may directly target these enzymes cannot be excluded. Long et al. recently confirmed that circPOSTN was overexpressed in glioma tissues and induced tumor cell proliferation by targeting the miR-361-5p/TPX2 axis (30). A second study showed that circPOSTN or TPX2 knockdown could inhibit HK2 expression levels, indicating that circPOSTN might be involved in glioma progression by affecting aerobic glycolysis (30). In parallel, Guan et al. demonstrated that silencing of circPITX1 impeded glycolysis and the radioresistance of glioma cells by absorbing miR-329-3p (40). Furthermore, circTTBK2, circEPHB4 and circ_0002755 have been reported to regulate the glycolytic process by sponging and sequestering miRNAs (43, 52, 165, 166). Therefore, inhibition of aerobic glycolysis may be a promising anti-tumor therapy. However, studies on metabolism-related circRNAs in glioma are still limited and further investigation is warranted.

### 4.5 CircRNA and Blood-Tumor Barrier

Blood-tumor barrier (BTB) is the result of heterogeneously disruption of BBB in the progression of tumor (167). BTB seriously hinders the delivery of the antineoplastic agents to brain tumor tissue and thus affects glioma curative efficacy. Interestingly, a recent study showed that circRNA DENND4C enhances BTB permeability by reducing tight junction-associated protein expression *via* sponging miR-577 as a ceRNA (80). This process effectively allows the anti-tumor drug doxorubicin (DOX) to pass through BTB and induce glioma cell apoptosis.

CircRNA_001160, derived from linear RNAANKRD17 cyclization, acts as an endogenous sponge for miR-195-5p to upregulate *ETV1*, thus diminishing BBB permeability. Overexpression of *ETV1* in GECs can upregulate tight junction-related proteins expression through binding to their promoter regions (79). Circ-USP1 has also been reported to modulate the permeability of the BTB through tight junctions, which is mediated by the miR-194-5p/FLI1 axis. Knockout of circ-USP1 reduces the expression of tight junction-related proteins, including claudin-5, occludin and ZO-1, in GECs, effectively promoting the anti-tumor effect of DOX (81).

### 4.6 Chemotherapeutic and Radiation Resistance

TMZ is a first-line chemotherapeutic drug for treating glioma and is usually routinely administered to high-graded glioma patients (WHO grade III-IV), especially GBM, following surgery to prevent glioma recurrence and prolong patient survival. However, chemoresistance is a major contributor to poor clinical efficacy (168). Recent studies found that circ_0005198, circASAP1 and hsa_circ_0072083 can induce TMZ resistance of glioma cells *via* miR-198/TRIM14, miR-502-5p/NRAS and miR-1252-5p/NANOG pathway, respectively (32–34). Additionally, circ_0072083 and hsa_circ_0042003 could be detected in exosomes and exosomal circ_0072083/hsa_circ_0042003 can increase TMZ resistance and act as promising therapeutic targets in glioma (34, 36). Moreover, downregulation of hsa_circ_0076248 or upregulation of its binding miR-181a not only suppressed cell proliferation and migration but also significantly increased the sensitivity of glioma cells to TMZ (46). Furthermore, miR-181b is also a potential therapeutic target for glioma chemoresistance because it regulates the sensitivity of gliomas to TMZ by targeting BCL-2 and EGFR (169, 170). In addition, hsa_circ_0110757 showed high expression in TMZ-resistant glioma cells, and knock down of hsa_circ_0110757 enhanced the chemosensitivity of glioma cells to TMZ *via* targeting miR-1298-5p/ITGA1 (35).

It should be mentioned that CSCs have the potential for self-renewal, proliferation, migration, invasion and differentiation. They can also become dormant to escape harmful stress. The primary or metastatic lesions may differentiate and proliferate when the harmful pressure is eliminated and subsequently disseminate to other tissues and organs, resulting in tumor relapse or metastasis (171, 172). EMT can mediate drug resistance by inducing transcription factors like twist and snail to bind to the promoter of ATP-binding cassette (ABC) transporters, which is responsible for the drug efflux by transporting drug to the extracelluar compartment. Therefore, circRNAs that can mediate cell stemness and EMT may participate in the drug resistance of tumor cells. More studies are needed to explore this mechanism. However, circRNA provides promising new therapeutic insights to address the chemoresistance of gliomas.

Radiation-induced damage to the glioma microenvironment may generate a tumor-susceptible niche and facilitate invasion and migration of residual glioma cells, contributing to disease relapse. CircATP8B4 and circCPA4 are overexpressed in glioma and reduce radiation sensitivity of glioma cells by serving as sponges for miR-766 and miR-760, respectively (38, 120). Studies have also shown that circ_VCAN expressed at a higher level in radioresistant gliomas cells compared to sensitive cells and overexpression of circ_VCAN accelerate carcinogenesis and reduce the radiosensitivity of gliomas by regulating miR-1183 (39). However, the AKT3-174aa protein encoded by hsa_circ_0017250 can enhance the glioma cell radiosensitivity by inhibiting the phosphorylation of AKTT308.

## 5 Clinical Applications in Glioma

Recently, a research team has found a novel approach to express protein through engineered circRNA vectors (173). This results showed that exogenous circRNA could express qualified proteins efficiently, indicating that circRNA is a promising molecule that could be applied to proteins expression field. It is worth noting that this study was conducted *in vitro* and deliver circRNA by cationic lipid transfection. However, the delivery of circRNA *in vivo* is more challenging. Meganck et al. (174) designed tissue-specific circRNA expression vectors using the recombinant *adeno-associated virus* (*AAV*) vector. In addition, they also found that these novel vectors could effectively express and translate proteins in mice. Therefore, some circRNA-loaded biological vectors could be applied in clinical therapy. More stable and specific vectors that suitable for circRNA delivery *in vivo* and *in vitro* still need to be studied.

In addition, some nucleic acid therapies for ncRNA should also be concerned. For example, antisense oligonucleotides (ASOs) are a class of single-stranded deoxyribonucleotides (15–20 nucleotides) that could bind to ncRNA through complementary sequences and inhibit its activity by promoting RNaseH degradation (175). Several studies have validated that ASO can inhibit the expression of ncRNA (e.g., lncRNA, miRNA), thus slowing down the tumor progression in mice (176, 177). A recent study utilized ASO (linear RNA ASO) to identify the biosynthetic way of ciRS-7 (CDR1as) is back-splicing pathway rather than intra-lariat pathway (178). However, appropriate designed ASO targeting intron sequences or back-splicing junction in the pre-mRNA can efficiently knock down ElciRNA or ciRNA (95, 96). Notably, circRNA ASO (only designed to target the circular junction) transfection would decrease the nascent circRNA level but would not impair transcription activity of the encoded gene (179).

In short, circRNA with tumor inhibitory effect can express proteins through vectors, while circRNA with carcinogenic effect can be used for clinical treatment with nucleic acid therapy. We believe that circRNA will make great progress in clinical treatment of gliomas in the future.

## 6 Discussion and Prospects

The mystery of circRNAs in glioma is now slowly being unraveled due to the arrival of high-throughput sequencing and algorithms. The temporal and spatial specificity of circRNA implies that circRNA is involved in the process of tumorigenesis and development. As a stage-specific indicator of tumorigenesis and development, circRNA has in-depth research significance and is worth further exploring. The expression abundance of circular RNA in the brain is higher than that of other organs, and it increases continuously from embryonic to adulthood to regulate brain development and functions related to neuroplasticity. CircRNAs levels change along with angiogenesis, autophagy, apoptosis, tumorigenesis and inflammation, and are closely related to brain tumors. Recently, increasing evidences advocated that circRNAs can mediate the occurrence and progression of glioma through miRNA sponging, transcriptional regulation and protein interactions. However, newly discovered translatable circRNAs have guided a novel and promising research direction. Scientists are currently studying how to associate these tumor-suppressing proteins with vaccines to benefit clinical treatment.

CircRNAs possess the potential as biomarkers for glioma diagnosis or follow-up due to their properties (high stability and long half-life). Mounting evidences have indicated that circRNAs may associate with WHO classification and prognosis in glioma. However, the current detection of circRNAs is predominantly carried out in tissues and cell lines. Liquid biopsy, a less invasive method, is limited in the clinical environment. The low abundance of circRNA makes it difficult to be accurately detected and identified. Therefore, we must improve the detection methods to improve the accuracy of detecting annular RNA. It is believed that in the near future, circRNA may be combined with traditional methods for early therapeutic evaluation with high sensitivity.

Aberrantly expressed circRNAs in glioma play vital roles in tumorigenesis through the circ-miRNA-mRNA axis and cancer signaling pathways. Additionally, the crosstalk of circRNAs with critical TME components contributes to tumorigenesis, progression and chemo- and radioresistance, in which the role of circRNAs in modulating tumor growth by regulating glycolytic metabolism should be emphasized. Moreover, there is growing evidence suggesting that circRNAs are implicated in chemo- and radioresistance of glioma cells. There is no doubt that circRNA will open up a new era for anti-tumor therapy. However, how to effectively deliver circRNA molecules to the target is the biggest problem facing their clinical application. Beyond that, circRNA may also participate in the immune response and could be a potential immunotherapy target, because circRNAs in EVs could act as tumor antigens to activate anti-tumor immune response based on the theory of circRNA binding to miRNA and protein.

Taken together, the current understanding of circRNA is just the tip of the iceberg. Considerable work is urgently needed to overcome the difficulties in the clinical applications for circRNA, such as high expense, difficulty of purification, ambiguous mechanism and the existence of secondary structure.

## Author Contributions

XZ conceived and designed the idea. MC wrote the major part of the manuscript. CY participated in manuscript writing and revision process of the manuscript. All authors contributed to the article and approved the submitted version.

## Funding

This study was funded by National Natural Science Foundation of China (No.81802760, 81702402), Science and Technology Project of Liaoning (No.20170520027), and 345 talent plan project of Shengjing Hospital (XZ, Lei Liu).

## Conflict of Interest

The authors declare that the research was conducted in the absence of any commercial or financial relationships that could be construed as a potential conflict of interest.

## Publisher’s Note

All claims expressed in this article are solely those of the authors and do not necessarily represent those of their affiliated organizations, or those of the publisher, the editors and the reviewers. Any product that may be evaluated in this article, or claim that may be made by its manufacturer, is not guaranteed or endorsed by the publisher.
